# Effects of Layered Nanoclays on the Cellular Structure, Dynamic–Mechanical–Thermal Properties and Fire Behavior of Flame-Retardant ABS Foams

**DOI:** 10.3390/polym17243285

**Published:** 2025-12-11

**Authors:** Marcelo Antunes, Farnaz Ghonjizade-Samani, Vera Realinho

**Affiliations:** Poly2 Group, Department of Materials Science and Engineering, Terrassa School of Industrial, Aerospace and Audiovisual Engineering (ESEIAAT), Technical University of Catalonia (UPC BarcelonaTech), C/Colom 11, 08222 Terrassa, Spain

**Keywords:** ABS foams, phosphorus-based flame retardants, montmorillonite, layered double hydroxide, cone calorimeter

## Abstract

The present work deals with the preparation and characterization of fire-retardant acrylonitrile–butadiene–styrene (ABS) foams incorporating 25 wt% of a phosphorus flame-retardant (PFR) system formed by 50% of ammonium polyphosphate (APP) and 50% of aluminum diethylphosphinate (AlPi). To further enhance performance, 5 wt% of the PFR was replaced by either montmorillonite (MMT) or layered double hydroxide (LDH) nanoparticles, maintaining the overall FR content constant. The formulations were prepared by melt blending, and foams were produced using a one-step supercritical carbon dioxide (sCO_2_) dissolution foaming process. The incorporation of the PFR, alone or partially replaced by nanoclays, resulted in foams with smaller cell sizes and higher cell nucleation densities compared to pure ABS, with cell sizes reducing from 60 μm to as low as 40 μm and cell densities reaching values > 10^7^ cells/cm^3^. The presence of LDH notably modified the thermal decomposition of ABS–PFR, increasing the temperature at 5% mass loss (T_5%_) by more than 10 °C and the amount of formed residue by more than 15%. The ABS–PFR/LDH foam also showed a higher glass transition temperature (3 °C increase) and a higher specific storage modulus (920 MPa·cm^3^/g, a more than 40% increase). Cone calorimetry revealed a very significant reduction in the peak of the heat release rate (PHRR) and increased residue formation for ABS–PFR compared to ABS (from 1672 kW·m^−2^ to as low as 483 kW·m^−2^). LDH nanoparticles further decreased HRR during the early quasi-static combustion stage of foams, indicating a more effective condensed-phase flame-retardant action than MMT.

## 1. Introduction

Acrylonitrile–butadiene–styrene (ABS) is one of the most used engineering polymers due to its good combination of properties and low cost, being vastly used in industrial sectors such as the automotive (interior and exterior car parts), building and construction (molded housings and small house appliances), as well as in electrical and electronic applications (for instance in telephone and computer housings), due to its high thermal insulation performance and the fact that its electrical properties do not change significantly with temperature and humidity [1]. Nevertheless, ABS is highly flammable, generating gases and toxic fumes during combustion [2]. Traditionally, the enhancement of ABS’s flame retardancy has been associated with the use of brominated flame retardant additives, vastly introduced in the 1960s and 1970s and very effective even at low concentrations [2]. However, the use of these halogenated flame retardants was demonstrated in the 1990s to adversely affect the environment and their use has been highly limited due to recent European environmental restrictions [3]—in some cases even resulting in their removal from the market, as in the case of octabromodiphenyl oxide [4]—making it critical to find alternative halogen-free flame retardant formulations.

Although ABS is a non-charring polymer, halogen-free phosphorous-based flame retardants (elemental red phosphorous, phosphines, phosphonium compounds, phosphonates, phosphites, phosphinates and phosphates) are inherently less efficient as they mostly act in the condensed phase by modifying the pyrolytic decomposition of the polymer and reducing the amount of combustible gases [5]; however, they are still the most used alternative to halogen-based flame retardants (FRs) [6], especially when combined with other FRs that promote the formation of an effective char layer, such as pentaerythritol or mannitol, particularly in intumescent formulations [7], expandable graphite or products derived from biomass [8], among others. There is also a significant interest in the use of FR systems containing layered nanoparticles, especially nanoclays [9,10]. Nanoclays have been shown to change the degradation pathway of ABS from β-scission (chain-end and middle) to a combined recombination and random scission, resulting in a reduction in the peak of the heat release rate obtained using a cone calorimeter from 20% to as high as 50% [2], as well as reducing material dripping during burning. Nevertheless, as nanoclays alone have been shown to still lack the required flame retardancy, research has indicated that systems combining low amounts of these layered nanoparticles with more traditional flame retardants are required to achieve effective flame retardancy [9,10].

Furthermore, there is growing interest in industrial sectors—particularly the automotive industry—in the replacement of conventional materials with lighter and more sustainable alternatives, as weight reduction not only improves fuel efficiency but also contributes to lower carbon emissions and enhanced overall energy performance. In this sense, due to their ease of processing and ease of cellular structure control, styrenic-based foams, and particularly ABS foams, have experienced great development, especially in terms of achieving microcellular or even nanocellular structures using foaming processes such as the MuCell injection-molding physical foaming process or the supercritical gas dissolution batch foaming process [11,12,13,14,15,16,17], hence achieving the best combination of weight reduction and mechanical performance.

In the pursuit to develop halogen-free flame-retardant ABS foams with optimized structural and functional performance, this work investigates how the incorporation of layered nanoclays affects the cellular morphology, thermal stability, dynamic–mechanical–thermal response, and fire behavior of ABS foams containing phosphorus-based flame retardants (PFRs). With this objective in mind, ABS was formulated with PFRs, either on their own or combined with two different types of layered nanoclays: montmorillonite (MMT), a cationic silicate, or hydrotalcite, a layered double hydroxide (LDH), and foamed by supercritical CO_2_ (sCO_2_) dissolution. The study explores the potential synergistic effects between PFRs and said nanoclays in simultaneously enhancing the flame retardancy and the specific mechanical performance of ABS foams, helping to close the research gap in terms of developing sustainable non-toxic halogen-free FR alternatives that not only do not compromise the mechanical performance of the foamed material, but even promote mechanical enhancements.

## 2. Materials and Methods

### 2.1. Materials

An acrylonitrile–butadiene–styrene copolymer (ABS), commercially named Elix 128 IG, was kindly provided by Elix Polymers (Tarragona, Spain) in pellet form. According to the manufacturer, the ABS contains 26–28 wt% of butadiene and has a melt volume rate of 15 cm^3^/10 min, measured at 220 °C under a 10 kg weight load (ISO 1133 [18]).

The base flame-retardant system consisted of ammonium polyphosphate (APP, Exolit AP422) and aluminum diethylphosphinate (AlPi, Exolit OP1230), both supplied as white powders by Clariant Produkte (Sulzbach, Germany). APP, with the chemical formula (NH_4_PO_3_)_n_, has a polymerization degree (n) higher than 1000, a phosphorus and nitrogen content of 31–32 wt% and 14–15 wt%, respectively, a density of 1.90 g·cm^−3^, and an average particle size of 15 µm. AlPi, with the chemical formula [(C_2_H_5_)_2_PO_2_]_3_Al, has a phosphorus content of 23.3–24.0 wt%, a density of 1.35 g·cm^−3^, and an average particle size of 30 µm.

Layered nanoparticles were employed as potential synergistic flame retardants. Specifically, montmorillonite (MMT, Cloisite SE3000), provided by BYK Additives (Geretsried, Germany) and organically modified with dimethyl-distearyl ammonium chloride, has an average particle size < 10 µm and a density of 1.3 g·cm^−3^. Additionally, a layered double hydroxide (LDH, Sorbacid 944) supplied by Clariant Produkte (Sulzbach, Germany) was used, with average Al, Mg, and Zn contents of 10, 15, and 11 wt%, respectively, a density of 2.21 g·cm^−3^, and an average particle size < 10 µm.

To promote dispersion of the phosphorus flame retardants and nanoparticles, a commercial dispersant additive (D, BYK P 4102) from BYK Additives (Geretsried, Germany) was incorporated.

All chemicals were used without any further purification.

### 2.2. Preparation of the Formulations

The formulations were prepared by melt-compounding the different components using a Brabender Plasti-Corder Lab-Station (W50 EHT internal mixer, Brabender GmbH & Co., KG, Duisburg, Germany) at a constant temperature of 160 °C and screw speed of 30–60 rpm, during a total mixing time of 12 min. Prior to compounding, ABS was dried at 80 °C for 4 h, and the phosphorus flame retardants (PFR) and the synergistic additives (MMT and LDH) were pre-dried at 100 °C overnight. Table 1 presents the compositions of the ABS formulations.

In order to prepare the precursors required for foaming (foaming precursors), the melt-blended formulations were quickly placed, after removing from the Brabender mixer, in a circular cavity mold with a diameter of 74 mm and a nominal thickness of 4 mm, and compression-molded by heating at 170 °C applying a constant pressure of 80 bar during 6 min using a hot-plate press IQAP LAP PL-15 (IQAP Masterbatch Group S.L., Barcelona, Spain). Subsequent cooling was performed under a constant pressure of 80 bar for 4 min using recirculating water.

### 2.3. Preparation of the Foams by sCO_2_ Dissolution

The foaming precursors were foamed with the use of supercritical carbon dioxide (sCO_2_) in a one-step process inside a high pressure vessel (CH-8610 Uster/Schweiz, Büchiglasuster, Switzerland). The foaming precursors were first saturated with sCO_2_ at 145 °C and 160 bar during 30 min. In the case of the precursors without flame retardants (i.e., ABS), the temperature was reduced to 110 °C (pressure at said temperature: 160 bar) in order to avoid deformation of the sample during the application of a sudden pressure drop until atmospheric pressure. Meanwhile, the precursors containing PFR, PFR/MMT and PFR/LDH were directly foamed at the saturation temperature and saturation pressure by applying a sudden release of pressure until atmospheric pressure.

### 2.4. Characterization

The density of both unfoamed and foamed ABS-based formulations was measured according to ISO 845 [19]. The cellular structure of the foams was analyzed by means of scanning electron microscopy (SEM) using a JEOL JSM-5610 (Tokyo, Japan) microscope. Samples were prepared by cryogenic fracture using liquid nitrogen and sputter depositing at their surfaces a thin layer of gold with a BAL-TEC SCD005 Sputter Coater under argon atmosphere. The average cell size (*ϕ*) and cell nucleation density (*N*_0_) were determined using the intercept counting method [20]. Two cell sizes were determined according to the direction of foam growth: *ϕ_VD_*, corresponding to the vertical growth direction (VD) and *ϕ_WD_*, corresponding to the width direction (WD). The cell aspect ratio (AR) was determined as the ratio between both cell sizes; i.e., AR = *ϕ_VD_*/*ϕ_WD_*. The cell nucleation density, which represents the number of cells per volume of unfoamed material, was determined assuming an isotropic distribution of spherical cells according to the following expression:

(1) $$N_{0}=\;{\left(\frac{n}{A}\right)}^{3/2}\left(\frac{\rho_{s}}{\rho }\right)$$

where *n* is the number of cells in the micrograph, *A* is its area (in cm^2^), and *ρ_s_* and *ρ* the density of the unfoamed and foamed material, respectively. A minimum of three micrographs were used per formulation in the analysis.

The thermal stability of the unfoamed and foamed materials was evaluated by thermogravimetric analysis (TGA) using a TGA/DSC1 Star System analyzer (Mettler-Toledo S.A.E., Barcelona, Spain). Samples of approximately 10.0 mg (unfoamed) and 7.0 mg (foamed) were heated from 30 to 1000 °C at a constant rate of 10 °C/min under a nitrogen flow of 30 ml·min^−1^.

Dynamic–mechanical–thermal analysis (DMTA) was carried out to evaluate the viscoelastic behavior of the unfoamed and foamed materials, focusing on their storage and loss moduli (E′ and E″), as well as the glass transition temperature (T_g_) of the SAN phase present in ABS. Measurements were performed using a DMA Q800 (TA Instruments, New Castle, USA) in single-cantilever mode, from 30 to 150 °C at 2 °C·min^−1^, with a dynamic strain of 0.1% and a frequency of 1 Hz. Rectangular specimens (35.5 × 12.5 × 3.5 mm^3^) were tested.

The heat release rate (HRR) was measured using an INELTEC cone calorimeter (INELTEC Barcelona, Spain) following the ISO 5660 standard [21]. Circular unfoamed and foamed specimens (74.0 ± 0.5 mm diameter, 3.5 ± 0.5 mm thickness) were exposed to a constant heat flux of 35 kW·m^−2^. From the HRR–time curves, key fire behavior parameters—including time to ignition (TTI), peak of the heat release rate (PHRR), time to PHRR (t_PHRR_), and total heat emitted (THE)—were obtained. The effective heat of combustion (EHC) was calculated by dividing THE by the sample’s mass loss.

## 3. Results

### 3.1. Cellular Structure of ABS-Based Foams

**Figure 1:**
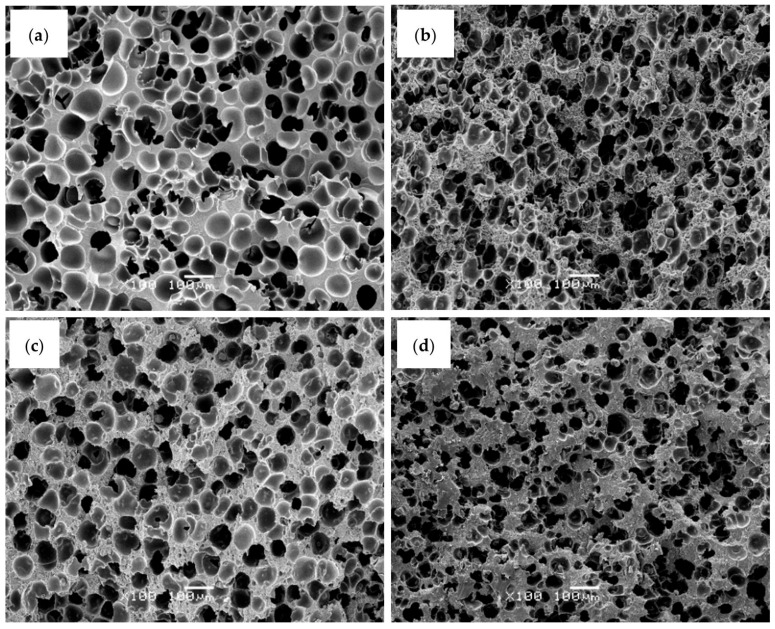
Characteristic SEM micrographs at ×100 magnification showing the cellular structure of ABS-based foams: (**a**) ABS, (**b**) ABS-PFR, (**c**) ABS-PFR/MMT, and (**d**) ABS-PFR/LDH.

In terms of cellular morphology, the incorporation of PFR, either alone or in combination with MMT or LDH, resulted in foams with slightly smaller cell sizes and higher cell nucleation densities compared to the pure ABS foam (see representative SEM micrographs in Figure 1 and corresponding parameters in Table 2).

As shown by the data presented in Table 2, ABS foams containing PFR, alone or in combination with MMT or LDH, exhibited higher relative densities than the pure ABS foam. This behavior can be attributed to a greater restriction to foam expansion caused by the presence of both PFR particles and layered nanoparticles. These features are consistent with their higher relative density and the heterogeneous cell nucleation promoted by the dispersed inorganic particles. Among the flame-retardant foams, those containing MMT or LDH showed cell aspect ratios closer to 1.0, indicative of a more homogeneous and isotropic cellular structure. In particular, the addition of LDH produced foams with slightly smaller cell sizes and higher cell densities than the MMT-containing foams, suggesting a more compact cellular morphology, although the overall nucleating effect of LDH was moderated by the relatively high total loading of PFR particles.

### 3.2. Thermal Stability

The thermal stability of the precursors and foams of ABS and ABS flame retardant formulations was studied by thermogravimetric analysis. The data, including the temperatures corresponding to a 5% of mass loss (T_5%_) and to the maximum mass loss rate (T_peak_), mass loss rate (MLR) and mass loss (ML) of each thermal decomposition (TD) stage, as well as the total fraction of residue at 800 °C, is presented in Table 3.

**Figure 2:**
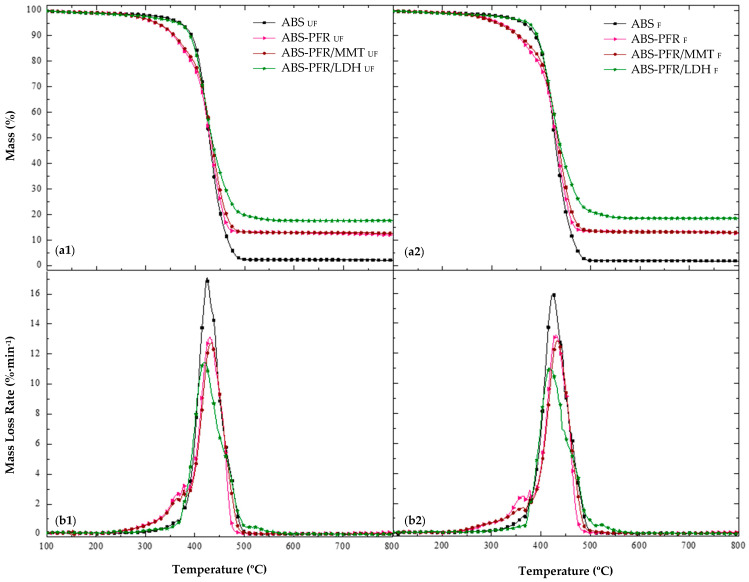
(**a**) TG and (**b**) dTG curves of unfoamed (**a1**,**b1**) and foamed (**a2**,**b2**) ABS-based formulations, obtained at 10 °C·min^−1^ using a nitrogen atmosphere.

As can be seen in Figure 2, ABS exhibited a characteristic one-stage degradation process, between approximately 300 °C and 500 °C, with T_5%_ and T_peak_ occurring at 370 °C and 425 °C, respectively. Additionally, as expected, it did not show significant residue formation, showing that it has no char ability on its own. It has been reported that ABS thermally decomposes by chain scission, butadiene decomposition starting earlier and finishing later than the styrenic part [22,23]. It should also be mentioned that a small peak of mass loss rate at 377 °C, attributed to the thermal degradation of the dispersant additive, was detected for all formulations.

All flame-retardant ABS formulations decomposed in two main stages. ABS-PFR and ABS-PFR/MMT exhibited identical decomposition patterns, showing a small decomposition step prior to the main thermal degradation step of ABS. Meanwhile, ABS-PFR/LDH displayed a second minor step after the main thermal decomposition step of ABS.

In the ABS-PFR formulation, the combination of APP and AlPi increased the temperature of the maximum mass loss rate (T_peak_) of ABS by 4.7 °C, with an additional small degradation stage occurring before that of neat ABS, as previously mentioned. This first stage started at approximately 230 °C and ended at 385 °C, with a mass loss of 17.5% and a T_peak_ of 367 °C. This formulation also exhibited an earlier onset of decomposition, with a T_5%_ approximately 50 °C lower than that of ABS, attributed to the thermal degradation of the PFR additives. As mentioned before, ABS-PFR/MMT showed a thermal stability similar to that of ABS-PFR, with only a slight increase in the residue at 800 °C and a higher T_peak_ corresponding to the mass loss of ABS.

The most significant differences in the thermal degradation profiles were observed for the ABS-PFR/LDH system. This formulation did not show the mass-loss stage typically associated with the thermal decomposition of APP/AlPi; instead, a new stage appeared at higher temperatures, between 500 °C and 550 °C, with a T_peak_ of 519 °C and a mass loss of 1.8%. In addition, a higher amount of residue at 800 °C was recorded for this material (Table 3). These findings suggest that LDH interfered with the conventional decomposition pathway of APP/AlPi [23], suppressing the release of volatile phosphorus species before the degradation of the ABS matrix and promoting instead a predominant action in the condensed phase. This behavior is consistent with previous reports indicating that the metal hydroxides and oxides generated during LDH decomposition can react with phosphoric acid derivatives, thereby retaining a greater fraction of phosphorus in the condensed phase and enhancing char formation [24,25].

In general, all foams exhibited similar degradation patterns to their unfoamed counterparts, with most foams showing a reduction in T_peak_ associated with ABS decomposition and comparable MLR and ML values. However, the ABS-PFR/LDH foam displayed a higher T_5%_ (374.0 °C) than its unfoamed counterpart (364.9 °C), an increase of approximately 9 °C. This behavior seems to be specific to the LDH-containing formulation and may be attributed to the synergistic effect of LDH in the condensed phase, as discussed earlier, in combination with the more compact cellular morphology of the ABS-PFR/LDH foam, characterized by its higher cell nucleation density and smaller cells, which likely contributed to delaying the onset of mass loss due to a more effective heat insulating effect.

### 3.3. Dynamic–Mechanical–Thermal Analysis (DMTA)

**Figure 3:**
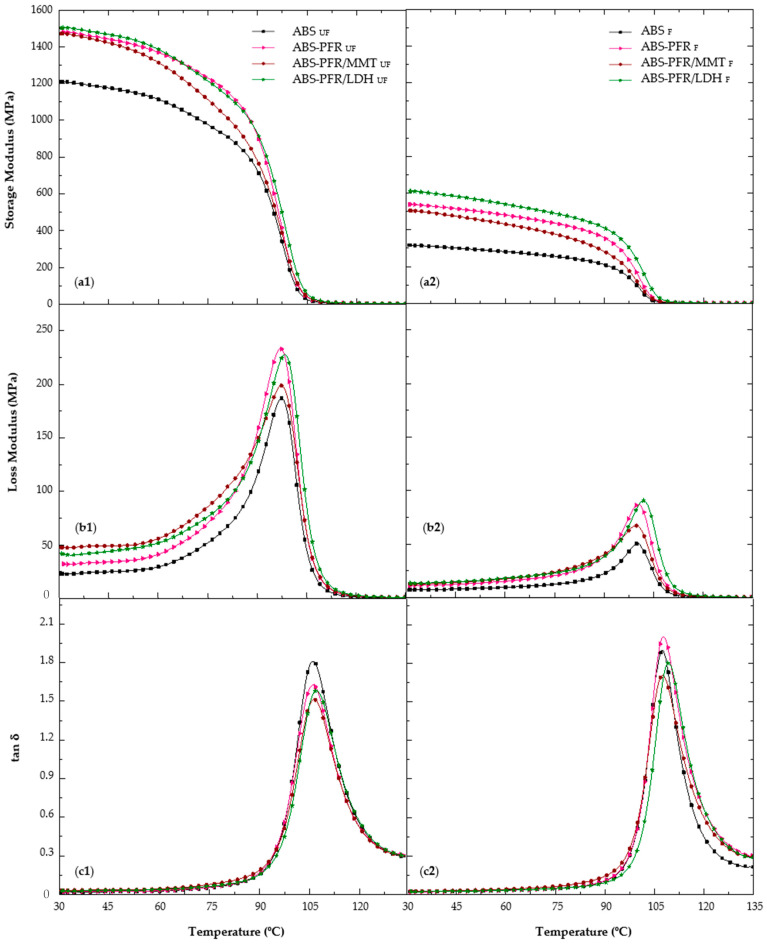
(**a**) Storage modulus, (**b**) loss modulus and (**c**) tan δ of unfoamed (**a1**–**c1**) and foamed (**a2**–**c2**) ABS-based formulations.

Figure 3 shows the storage modulus (E′), loss modulus (E″), and tan δ curves from 30 to 135 °C for the unfoamed and foamed ABS and ABS flame-retardant formulations.

In Figure 3, it is possible to observe a first zone, between 30 °C and 90 °C, where the materials gradually reduce their stiffness due to an increase in their molecular relaxations. This fact could be related to a certain degree of molecular chain movement of the rubbery part of ABS. Between 90 °C and 110 °C, when the primary transition of styrene-acrylonitrile (SAN) [26,27,28,29] occurs, the material’s molecular movement and energy dissipation strongly increased, as can be seen from the decrease in the storage modulus (E′), and the increase in the loss modulus (E″) and tan δ. Once this transition was surpassed, a zone of high viscoelasticity, where the elastic part of the material exceeds its recovery capacity, took place [28,29].

From Table 4, it is possible to see that the specific storage modulus (E′/ρ; i.e., the storage modulus divided by the density of the material) of the unfoamed ABS-PFR was 19% higher than that of ABS, with this improvement being even higher for the foamed material (30%). Both values are presented in column “ΔE′/ρ”, which represents the variation in the specific elastic modulus of ABS-PFR materials regarding the specific elastic modulus of ABS. No significant differences were noticed for the unfoamed materials when replacing 5 wt% of PFR with layered nanoparticles (MMT or LDH). Nevertheless, an increase of 40% in the specific storage modulus was observed for the ABS-PFR/LDH foam when compared to the ABS foam, indicating a positive effect of foaming on the specific storage modulus of this material.

No important changes in the glass transition temperature were observed in the presence of the PFR and layered nanoclays in the unfoamed materials. Nevertheless, a slight increase in the T_g_ (3 °C) was noticed for the ABS-PFR/LDH foam when compared to ABS. This observation could indicate a positive interference of the foaming process in the dispersion and/or exfoliation of LDH nanoparticles, leading to a better interaction between these nanoparticles and the polymer matrix.

Due to an increase in the elastic contribution caused by the presence of the flame retardant additives, a decrease in the tan δ peak intensity during glass transition was observed for the unfoamed materials. Furthermore, when nanoclays were added, the intensity of the loss modulus peak was reduced when compared to ABS-PFR, indicating that the presence of the layered nanoparticles slightly decreased the polymer chain mobility during the thermally activated process. For the foamed materials, this peak intensity was higher compared to the unfoamed counterparts. This could be explained by a decrease in the elastic contribution and an apparent increase in energy dissipation promoted by the microcellular foam structure.

### 3.4. Fire Behavior

**Figure 4:**
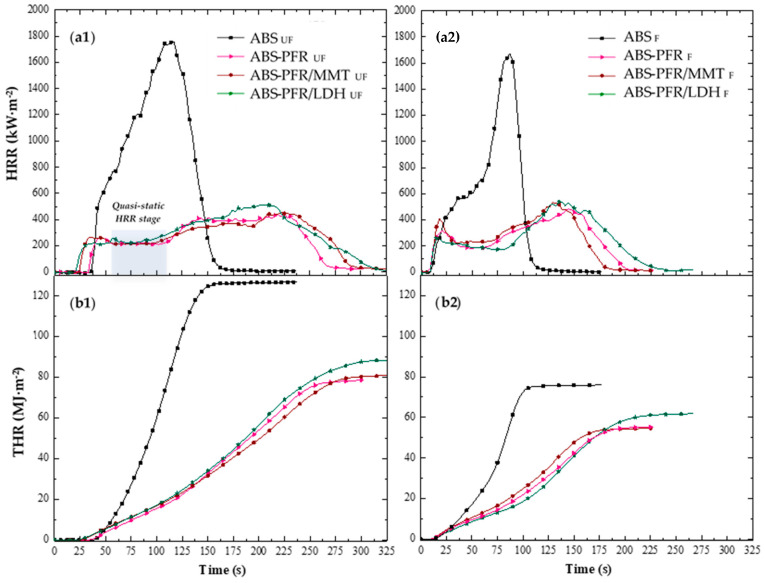
(**a**) Heat release rate (HRR) and (**b**) total heat released (THR) as a function of time for the unfoamed (**a1**,**b1**) and foamed (**a2**,**b2**) ABS-based formulations. In (**a1**), the quasi-static HRR stage is observed for all formulations, although only that of the ABS-PFR formulation (shaded region) is highlighted for clarity.

Figure 4 illustrates the characteristic heat release rate (HRR) curves of the unfoamed and foamed materials, and Table 5 summarizes the main results obtained from their analysis. As expected, ABS showed a fire behavior typical of non-charring polymers [30,31,32]. The HRR increased quickly after ignition, as a result of the rapid flame spread, followed by a less intense increase period until reaching its maximum value (PHRR).

Improvements in flame retardancy were observed upon the incorporation of APP/AlPi into ABS. Specifically, reductions in the peak of the heat release rate (PHRR) and the total heat evolved (THE), together with an extension of the burning time, were recorded. Moreover, for the ABS-PFR sample, a quasi-static HRR stage (marked in grey in Figure 4(a1)) was detected during the early stages of combustion (approximately between 55 and 110 s), which may be attributed to the formation of a carbonaceous and/or residual layer on the surface of the sample. It is well established that APP acts as a char promoter in various polymer matrices, leading to the formation of a crosslinked structure on the material’s surface that behaves as a physical barrier [32,33]. Meanwhile, AlPi has been reported to inhibit flame propagation through the release of phosphorus radicals, which can act as scavengers of HO· and H· radicals yielded during polymer combustion [34,35]. This latter effect is consistent with the greater reduction in the effective heat of combustion (EHC) observed for the ABS-PFR sample. However, the subsequent increase in HRR after the quasi-static stage indicates that the formed protective layer was not fully effective in preventing mass and heat transfer.

The incorporation of layered MMT nanoparticles decreased the HRR after the quasi-static stage of ABS-PFR, indicating an enhanced residue-forming effect and, consequently, an improvement in the barrier performance. Some studies have reported that during combustion these layered nanoparticles tend to migrate to the material’s surface, improving the fire resistance by promoting the formation of a more cohesive protective layer [36].

The presence of LDH particles promoted a higher residue content after the CC test, as well as increased EHC and PHRR values, as shown in Table 5. These findings suggest that the metal hydroxides within the LDH nanoparticles likely interacted with the decomposition products of the APP/AlPi system, promoting the retention of phosphate species in the condensed phase during the early stages of combustion. However, they also limited the release of phosphorus radicals into the gas phase, which is consistent with TGA results. Overall, these results indicate that the enhancement of the condensed-phase mechanism provided by the LDH nanoparticles is less significant than the reduction in the gas-phase mechanism observed for the ABS-PFR formulation.

By reducing the density of the ABS sample to less than half through foaming, it was possible to observe a decrease in the total heat emitted (THE), as well as in the time to ignition (TTI), time to PHRR (t_PHRR_), and burning time compared to the unfoamed ABS. This behavior was attributed to the lower polymer volume fraction and the presence of a cellular structure in the ABS foam. This difference in fire behavior is not surprising, since the fire performance of materials depends on the sample’s dimensions, density, and geometry, besides material’s thermal properties during combustion; i.e., thermal conductivity and heat capacity [37]. The ABS foam also showed a more rapid increase in HRR until reaching its peak (PHRR), which could be related to a post–cell structure collapse and a limited amount of polymer available for combustion.

The ABS flame-retardant foams exhibited shorter times to ignition and to PHRR (t_PHRR_) and overall burning durations compared to the unfoamed samples. Only minor differences were observed in the PHRR values of the char-forming materials between the unfoamed and foamed samples, with the flame-retardant effect of the PFRs prevailing in both cases. However, a gradual decrease in HRR during the quasi-static stage was observed for the LDH-containing foam compared to its unfoamed counterpart (as shown in Figure 4(a2)). This behavior could be related to a better dispersion and/or exfoliation of the nanoparticles in the microcellular foam, consistent with the higher T_g_ value, which promoted the formation of a more cohesive and protective char layer during this period. A slight difference in combustion time and THR evolution was also observed for the ABS-PFR/MMT foam (see Figure 4(b2)), which could be attributed mainly to its lower density when compared to the other flame-retardant foams rather than to interferences between different flame-retardant mechanisms.

## 4. Conclusions

Although the addition of nanoclays, both MMT and LDH, led to foams with generally smaller cell sizes and higher cell densities (reaching cell nucleation densities higher than 10^7^ cells/cm^3^), the effect of these nanoparticles in the cellular structure of ABS foams was only marginal, with a maximum cell size reduction of around 30%, explained by the high total concentration of added PFR particles.

In contrast to MMT, LDH nanoparticles interacted with the pyrolysis thermal decomposition of phosphorus flame retardant additives, enhancing the charring ability (more than 15% increase in the amount of formed residue) and improving the thermal stability of the ABS-PFR formulation (more than 10 °C increase in T_5%_).

ABS flame-retardant foams exhibited greater improvements in the specific storage modulus compared to their unfoamed counterparts, reaching specific values as high as 920 MPa·cm^3^/g, a more than 40% increase. The glass transition temperature of the SAN phase was not significantly affected by the presence of the PFR system (APP/AlPi) or by the incorporation of MMT or LDH nanoparticles. Nevertheless, the ABS-PFR/LDH foam showed a 3 °C increase in this transition temperature, which can be associated with an enhanced dispersion and/or partial exfoliation of the layered nanoparticles promoted by the foaming process.

Improved fire behavior was observed upon incorporating the PFR system into the ABS matrix, as evidenced by the substantial reductions in both PHRR and EHC (from 1672 kW·m^−2^ to 483 kW·m^−2^ and from 32 MJ·kg^−1^ to around 23 MJ·kg^−1^, respectively). The higher amount of residue in ABS-PFR/LDH compared to ABS-PFR (16.4 wt% vs. 11.7 wt%) suggests that the presence of LDH nanoparticles promoted an enhanced condensed-phase mechanism during combustion, consistent with the thermogravimetric analysis results. In comparison, foams exhibited significantly shorter ignition and combustion times as well as lower THE values than their unfoamed counterparts (30–40% reduction). Regarding PHRR, no significant differences were detected among the charring samples; however, a noticeable reduction in HRR during the quasi-steady stage at the initial phase of combustion was observed when LDH particles were present in the cell walls of ABS foams. This behavior indicates an improvement in the condensed-phase mechanism compared to the unfoamed counterpart, likely promoted by the combined effect of the foaming process and the more efficient dispersion of LDH nanoparticles.

## Figures and Tables

**Table 1:** Composition of the ABS-based formulations.

Material Code/Amount of Component (wt%)	ABS	D	APP	AlPi	MMT	LDH
ABS	98.0	2.0	-	-	-	-
ABS-PFR	73.0	2.0	12.5	12.5	-	-
ABS-PFR/MMT	73.0	2.0	10.0	10.0	5.0	-
ABS-PFR/LDH	73.0	2.0	10.0	10.0	-	5.0

**Table 2:** Relative density, average cell sizes, aspect ratio and cell nucleation density of ABS foams.

Material Code	Relative Density	*ϕ*_VD_ (μm)	*ϕ*_WD_ (μm)	AR	*N*_0_ (cells/cm^3^)
ABS	0.47	58 ± 17	60 ± 13	1.0	8.11 × 10^6^
ABS-PFR	0.57	54 ± 15	46 ± 15	1.2	1.14 × 10^7^
ABS-PFR/MMT	0.54	55 ± 16	57 ± 5	1.0	9.37 × 10^6^
ABS-PFR/LDH	0.58	39 ± 12	44 ± 8	0.9	1.24 × 10^7^

**Table 3:** TG and dTG results of unfoamed (UF) and foamed (F) ABS-based formulations.

Material Code	TD Step	T_5%_ (°C)		T_peak_ (°C)		MLR (%·min^−1^)		ML (%)		R_800°C_ (%)	
		UF	F	UF	F	UF	F	UF	F	UF	F
ABS	1	370.2	363.3	425.0	425.3	17.9	16.0	97.9	97.0	2.1	3.0
ABS-PFR	1	321.3	314.8	367.0	363.7	2.7	2.5	17.5	17.7	11.9	12.6
	2	-	-	430.3	429.8	13.2	13.2	70.6	69.7		
ABS-PFR/MMT	1	321.6	309.6	364.6	364.0	2.4	1.7	15.9	15.1	12.6	12.8
	2	-	-	434.3	431.7	12.8	12.8	71.5	72.1		
ABS-PFR/LDH	1	364.9	374.0	420.2	417.3	11.4	11.0	80.7	79.5	17.5	18.4
	2	-	-	519.0	523.1	0.5	0.6	1.8	2.1		

**Table 4:** DMTA results of unfoamed (UF) and foamed (F) ABS-based formulations.

Material Code	*E*’ at 30 °C (MPa)		*E*’/ρ at 30 °C (MPa·cm^3^/g)		Δ*E*’/ρ at 30 °C (%)		T_g_ (°C)		Peak of tan δ	
	UF	F	UF	F	UF	F	UF	F	UF	F
ABS	1149 ± 56	310 ± 9	1131	655	-	-	106.2 + 0.1	106.2 + 0.1	1.85 ± 0.05	1.88 ± 0.02
ABS-PFR	1498 ± 28	540 ± 2	1348	852	19.2	30.1	106.1 + 0.2	107.6 + 0.2	1.63 ± 0.01	2.00 ± 0.01
ABS-PFR/MMT	1472 ± 12	496 ± 12	1315	814	16.3	24.3	106.1 + 0.3	107.7 + 0.1	1.52 ± 0.01	1.70 ± 0.01
ABS-PFR/LDH	1513 ± 15	600 ± 13	1346	920	19.0	40.6	107.0 ± 0.1	109.3 ± 0.1	1.57 ± 0.02	1.81 ± 0.01

**Table 5:** Cone calorimeter results of unfoamed (UF) and foamed (F) ABS-based formulations.

Material Code	TTI (s)		PHRR (kW·m^−2^)		t_PHRR_ (s)		THE (MJ·m^−2^)		EHC (MJ·kg^−1^)		Residue (wt%)	
	UF	F	UF	F	UF	F	UF	F	UF	F	UF	F
ABS	36	9	1758	1672	117	87	126.7	76.0	32.6	31.9	0.3	0.2
ABS-PFR	35	7	440	483	225	144	78.4	55.3	22.0	23.4	11.9	11.7
ABS-PFR/MMT	24	5	455	532	222	129	88.3	54.7	24.1	24.5	12.7	12.3
ABS-PFR/LDH	23	5	515	537	204	135	93.5	61.9	28.5	28.0	16.6	16.4

## Data Availability

The original contributions presented in this study are included in the article. Further inquiries can be directed at the corresponding author.

## Abbreviations

The following abbreviations are used in this manuscript:

ABS	Acrylonitrile–butadiene–styrene
AlPi	Aluminum diethylphosphinate
APP	Ammonium polyphosphate
AR	Cell aspect ratio
CC	Cone Calorimeter
D	Dispersant additive
DMTA	Dynamic–mechanical–thermal analysis
EHC	Effective heat of combustion
F	Foamed
HRR	Heat release rate
LDH	Layered double hydroxide
E″	Loss modulus
ML	Mass loss
MLR	Mass loss rate
MMT	Montmorillonite
N_0_	Nucleation density
PFR	Phosphorus flame-retardant
Poly2	Polyfunctional polymeric materials
PHRR	Maximum value of heat release rate
SEM	Scanning electron microscopy
SAN	Styrene-acrylonitrile
E′	Storage modulus
sCO_2_	Supercritical carbon dioxide
TGA	Thermogravimetric analysis
T_5%_	Temperature corresponding to a 5% of mass loss
T_g_	Glass transition temperature
T_peak_	Temperature corresponding to the maximum mass loss rate
TD	Thermal decomposition
THE	Total heat evolved
THR	Total heat released
t_PHRR_	Time to peak heat release rate
TTI	Time to ignition
UF	Unfoamed
VD	Vertical foam growth direction
WD	Width foam growth direction
wt%	Weight fraction
